# Advocacy for Gender Affirming Care: Learning from the Injectable Estrogen Shortage

**DOI:** 10.1089/trgh.2017.0025

**Published:** 2018-03-01

**Authors:** Sophia Geffen, Tim Horn, Kimberleigh Joy Smith, Sean Cahill

**Affiliations:** ^1^ Department of Health Policy Research, The Fenway Institute, Fenway Health, Boston, Massachusetts.; ^2^ HIV and HCV Programs, Treatment Action Group, New York, New York.; ^3^ Community Health Planning and Policy, Callen-Lorde Community Health Center, New York, New York.; ^4^ Bouve College of Health Sciences, Northeastern University, Boston, Massachusetts.; ^5^ Department of Health Law, Policy and Management, Boston University School of Public Health, Boston, Massachusetts.

**Keywords:** public policy and advocacy, transgender, gender dysphoria, gender transition, clinical care

## Abstract

Hormone therapy is medically necessary for many transgender individuals. The U.S. Food and Drug Administration (FDA) and pharmaceutical companies' failure to guarantee a supply of injectable estrogen in 2016 and 2017 for transgender individuals is a violation of their right to comprehensive medical treatment, free of discrimination. A series of advocacy actions eventually led to all formulations of injectable estrogen being restored to market; however, long-term solutions to supply interruptions of injectable estrogen are needed. Long-term solutions should address the lack of federally funded research and, consequently, evidence-based practice on hormone therapy for gender affirmation.

## Introduction

Hormone therapy is a medically necessary intervention for many transgender individuals.^1^ Studies have shown that gender affirmation through hormone therapy can improve psychological adjustment and quality of life, including reduced anxiety and depression, higher self-esteem, and improved social functioning.^1–3^ The class of estrogen used for feminizing therapy is estradiol and is commonly delivered to transgender women through a patch, oral or sublingual tablet, or injection.^4^

There are no studies evaluating the benefits or risks of injectable estradiol, resulting in a lack of empirical data.^5^ Patients report that injectable estradiol may offer faster, earlier breast development (personal communication, Dr. Tim Cavanaugh [Director of Transgender Health] and Dr. Alex Gonzalez [Medical Director], Fenway Health, 2016). Patients who do not achieve adequate feminization or adequate circulating blood concentrations of estradiol through oral and transdermal estrogen are often referred to injectable forms.

## The Shortage of Injectable Estrogen

According to the U.S. Food and Drug Administration (FDA), a shortage of estradiol valerate began on August 10, 2016 and was fully resolved on June 12, 2017.^6^ However, the Callen-Lorde Community Health Center in New York City reported shortages in July 2016, when they received none of the 20 and 40 mg/mL formulations in their weekly drug shipment, and were told that the shortage would last until at least October 2016. This shortage affected the availability of Delestrogen (Parr Sterile Products) and its generic counterpart estradiol valerate (Perrigo), in the 10, 20, and 40 mg/mL formulations.^7^ Prescribing practices and patient preferences differ significantly through the country. However, providers and patients in Boston and New York City report that injectable estrogen at the 40 mg/mL dosage is the highest and most frequently prescribed dosage of injectable estrogen for transgender women.^8^

Reasons for the shortage were ill-defined, with the FDA listing “shortage of an inactive ingredient component” for Perrigo's product, and listing “other” as the cause of the shortage of Par's product, along with estimated release dates. Consumers and providers were not provided any information regarding what is required to resolve the shortage, and posted release dates often were not met. In response to the shortage, The Fenway Institute, Callen-Lorde, and Treatment Action Group published an issue brief and coauthored a sign-on letter with 570 signatures from physicians, public health professionals, advocates, and members of the LGBTQ community.^9^ The letter was delivered to Par and Perrigo Pharmaceuticals. Advocates met with FDA and submitted comment to the agency on off-label uses of approved medications.

Injectable estrogen is not the only recent critical shortage. As of March 1, 2018, 89 shortages are being reported by the FDA, including several emergency medicine drugs and the standard-of-care treatment for primary and secondary syphilis. The FDA should not merely reiterate vague, manufacturer-provided information regarding drug shortages. This lack of proactive leadership regarding stock-outs of essential drugs make it more difficult for healthcare consumers, including transgender people, to access comprehensive healthcare and clinicians to provide high-quality patient care.

The FDA stressed in a January 2017 meeting with community leaders that it is doing all it is statutorily allowed to do to address the estrogen shortage. Because drug components are proprietary, the FDA cannot specify publicly what specific component shortages or supply chain issues were causing the injectable estrogen shortage. In addition, it cannot force a manufacturer to produce more of a drug when a shortage occurs.

## Long-Term Solutions

The opaque drug shortage reporting practices by the FDA and by the manufacturers highlight the need for a more transparent and proactive process to ensure that medications are kept on the market. Either through legislation or regulations, the FDA's oversight of manufacturer reports should be strengthened to ensure that all agency communications provide clear information describing the reason(s) for the drug's shortage, manufacturing and regulatory steps taken to address the shortage, and accurate stock replenishment dates. In addition, the FDA should be more proactive in establishing contingency plans in the event of prolonged shortages, including expedited approval of bioequivalent products.

The FDA and the pharmaceutical companies both have an obligation to maintain a constant supply of approved treatments and products. The shortage of injectable estrogen caused a significant treatment interruption, increasing the risk of pharmacokinetic variability, attenuated effectiveness, and side effects. There was also significant concern among providers that patients would turn to street injections to replace what they were receiving in clinics, putting them at risk.^10^ Provider experience suggests that black market injectable estrogen is often diluted or laced with other potentially harmful ingredients. Individuals using street hormones may also share needles, risking exposure to HIV and hepatitis C. During the shortage, this concern was addressed with individual and community-level patient education and awareness.

In the last 10 years, many professional associations, including the American Medical Association, have issued statements or guidelines supporting effective treatment protocols for gender dysphoria.^11^ Review articles have been published in major peer-reviewed journals.^12–15^ Gender affirmation therapy has advanced since early treatment protocols were shown to be associated with high rates of thromboembolic disease. Over time, safer treatments and a wider range of options have been developed, including transdermal patches, oral/sublingual tablets, and injectable medications. However, there are no medications or other treatments that are FDA-approved for the purpose of gender affirmation.^16^ In contrast, there are over 17 estrogen medications with approved FDA labels for menopausal women.^17^

The FDA ensures the safety and efficacy of estrogen for menopausal, cisgender women through on-label use informed by clinical trials, but allows off-label use of estrogen for transgender women to remain the standard of care. Such disparate treatment is not acceptable. The FDA should acknowledge that use of injectable estrogen is common among transgender women and accept responsibility for ensuring correct prescribing and usage information. This will require agency engagement with manufacturers to prioritize registrational trials in support of package insert updates or expanded indications for transgender women. The FDA should also work with manufacturers of products used for masculinizing therapy among transgender men, notably testosterone.

## Conclusion

There is a pressing need for (1) clinical research in support of supplemental FDA approvals of injectable estrogen for transgender women; (2) safety, tolerability, efficacy, and acceptability comparisons with other estrogen-based formulations; and (3) emerging hormonal products with potential for transgender women. These all require the attention of the federal Department of Health and Human Services. NIH-funded research programs should help fill critical gaps in the clinical science of hormone treatment for gender affirmation. More research is required to strengthen standard-of-care clinical management guidance for transgender women, to support evidence-based policies and to safeguard against deprioritized and protracted disruptions in access, such as those associated with drug shortages.

## References

[1] Feldman J, Spencer K Chapter 18 Medical and surgical management of the transgender patient: what the primary care clinician needs to know. In: Fenway Guide to Lesbian, Gay, Bisexual, and Transgender Health 2nd Edition. (Makadon HJ, Mayer KH, Potter J, Goldhammer H; eds). Philadelphia: American College of Physicians, 2015, pp. 479–511

[2] Glynn TR, Gamarel KE, Kahler CW, et al. The role of gender affirmation in psychological well-being among transgender women. Am Psychol Assoc. 2016;3:336–344 10.1037/sgd0000171 PMC5061456 27747257

[3] Gómez-Gil E, Zubiaurre-Elorza L, Esteva I, et al. Hormone-treated transsexuals report less social distress, anxiety and depression. Psychoneuroendocrinology. 2012;37:662–670 21937168 10.1016/j.psyneuen.2011.08.010

[4] Deutsch MB Overview of feminizing hormone therapy. Center of Excellence for Transgender Health 2016 Available at: http://transhealth.ucsf.edu/trans?page=guidelines-feminizing-therapy Accessed 106, 2017

[5] Fenway Health. The medical care of transgender persons. Fenway Health 2015 Available at: www.lgbthealtheducation.org/wp-content/uploads/COM-2245-The-Medical-Care-of-Transgender-Persons.pdf Accessed 106, 2017

[6] U.S. Food & Drug Administration. Current and resolved drug shortages and discontinuations reported to FDA: estradiol valerate injection. FDA. 2017 Available at: https://www.accessdata.fda.gov/scripts/drugshortages/dsp_ActiveIngredientDetails.cfm?AI=Estradiol%20Valerate%20Injection,%20USP&st=r&tab=tabs-1 Accessed 106, 2017

[7] U.S. Food & Drug Administration. Current and resolved drug shortages and discontinuations reported to FDA: estradiol valerate injection. FDA. 2016 Available at: www.accessdata.fda.gov/scripts/drugshortages/dsp_ActiveIngredientDetails.cfm?AI=Estradiol%20Valerate%20Injection,%20USP&st=c Accessed 106, 2017

[8] Moore C Exclusive: there's an injectable estrogen shortage that's leaving trans women in Crisis. Out. 2016 Available at: www.out.com/out-exclusives/2016/8/04/exclusive-theres-injectable-estrogen-shortage-thats-leaving-trans-women Accessed 106, 2017

[9] Geffen S, Horn T, Smith KJ Resolving the current injectable estrogen shortage: a public health imperative. The Fenway Institute, Treatment Action Group, and Callen-Lorde Community Health Center 2016 Available at: fenwayhealth.org/wp-content/uploads/Resolving-the-current-injectable-estrogen-shortage-A-public-health-imperative.pdf Accessed 106, 2017

[10] HRC Staff. HRC Takes Action on Injectable Estrogen Shortage. Human Rights Campaign. 2016 Available at: https://www.hrc.org/blog/hrc-takes-action-on-injectable-estrogen-shortage Accessed 106, 2017

[11] Lambda Legal. Professional organization statements supporting transgender people in health care. Lambda Legal. 2013 Available at: https://www.lambdalegal.org/publications/fs_professional-org-statements-supporting-trans-health Accessed 35, 2018

[12] Spack NP Management of transgenderism. JAMA. 2013;309:478–484 23385274 10.1001/jama.2012.165234

[13] Kreukels BP, Cohen-Kettenis PT Puberty suppression in gender identity disorder: the Amsterdam experience. Nat Rev Endocrinol. 2011;7:466–472 21587245 10.1038/nrendo.2011.78

[14] Murad MH, Elamin MB, Garcia MZ, et al. Hormonal therapy and sex reassignment: a systematic review and meta-analysis of quality of life and psychosocial outcomes. Clin Endocrinol (Oxf). 2010;72:214–231 19473181 10.1111/j.1365-2265.2009.03625.x

[15] Olson J, Forbes C, Belzer M Management of the transgender adolescent. Arch Pediatr Adolesc Med. 2011;165:171–176 21300658 10.1001/archpediatrics.2010.275

[16] Fenway Health. The medical care of transgender persons. 2015 Available at: www.lgbthealtheducation.org/wp-content/uploads/COM-2245-The-Medical-Care-of-Transgender-Persons.pdf Accessed 106, 2017

[17] The U.S. Food and Drug Administration. Estrogen and estrogen with progestin therapies for postmenopausal women. FDA. 2016 Available at: www.fda.gov/Drugs/DrugSafety/InformationbyDrugClass/ucm135318.htm Accessed 106, 2017

